# Children and adults produce distinct technology- and human-directed speech

**DOI:** 10.1038/s41598-024-66313-5

**Published:** 2024-07-06

**Authors:** Michelle Cohn, Santiago Barreda, Katharine Graf Estes, Zhou Yu, Georgia Zellou

**Affiliations:** 1 Phonetics Laboratory, Department of Linguistics, University of California, Davis, Davis, USA; 2 Language Learning Lab, Department of Psychology, University of California, Davis, Davis, USA; 3 Natural Language Processing (NLP) Lab, Department of Computer Science, Columbia University, New York, USA

**Keywords:** Speech adaptation, Human–computer interaction, Anthropomorphism, Children, Human behaviour, Psychology

## Abstract

This study compares how English-speaking adults and children from the United States adapt their speech when talking to a real person and a smart speaker (Amazon Alexa) in a psycholinguistic experiment. Overall, participants produced more effortful speech when talking to a device (longer duration and higher pitch). These differences also varied by age: children produced even higher pitch in device-directed speech, suggesting a stronger expectation to be misunderstood by the system. In support of this, we see that after a staged recognition error by the device, children increased pitch even more. Furthermore, both adults and children displayed the same degree of variation in their responses for whether “Alexa seems like a real person or not”, further indicating that children’s conceptualization of the system’s competence shaped their register adjustments, rather than an increased anthropomorphism response. This work speaks to models on the mechanisms underlying speech production, and human–computer interaction frameworks, providing support for routinized theories of spoken interaction with technology.

## Introduction

We are in a new digital era: millions of adults and children now regularly talk to voice-activated artificially intelligent (voice-AI) assistants (e.g., Amazon’s Alexa, Apple’s Siri, Google Assistant)^1–3^. These interactions with technology raise novel questions for our understanding of human communication and cognition, particularly across the lifespan. The current study tests how adults and children talk to voice assistants, compared to when they are talking to another person. In particular, we examine whether adults and children differ in their voice-AI ‘registers’. A register is a systematic set of speech adjustments made for a category of context or interlocutor, such as the higher and wider pitch variation in infant-directed speech (“DS”)^4–7^. Register adjustments can be a window into speakers’ social cognition: people produce more effortful speech adaptations for listeners they think are more likely to misunderstand them (e.g., a non-native speaker^8,9^, computer system^10,11^), producing targeted adjustments (c.f., ‘Audience Design’^12–14^). When talking to technology, adults often make their speech louder and slower^15^; this is true cross-linguistically, including for voice assistants in English^15–18^ and German^19,20^, a robot in Swedish^21^, and computer avatar in English^10^, and it is consistent with the claim that people conceptualize technological agents as less communicatively competent than human interlocutors^11,15,22^. In some cases, English and French speakers also make their speech higher pitched when talking to another person compared to a voice assistant^17^ or robot^23^, respectively. Taken together, the adjustments observed in technology-DS often parallel those made in challenging listening conditions; in the presence of background noise, speakers produce louder, slower, and higher pitched speech^24,25^.

Do adults and children produce distinct speech registers when talking to people compared to technology? On the one hand, *media equivalence theories* propose that when a person detects a sense of humanity in a technological system, they automatically transfer human social rules and norms to the device (e.g., ‘Computers are Social Actors framework’^26,27^; ‘Media Equation theory’^28^). Broadly, these accounts signify a form of anthropomorphism, whereby people attribute human-like qualities (e.g., intention, agency, emotion) to living or nonliving entities (e.g., animals, wind, etc.)^29–31^. Indeed, there is some initial evidence of anthropomorphism of voice assistants: adults perceive their apparent gender^32,33^, emotional expressiveness^34^, and age^35^. The degree of ‘equivalence’ is also likely to vary developmentally. Children’s willingness to anthropomorphize (non-human) animate^36^ and inanimate objects^37^, as well as have imaginary ‘friends’^38,39^, is well-documented in the literature. Children also engage with technology in a qualitatively different manner from adults^40^. For example, in a study of YouTube videos, children regularly asked voice assistants personal questions (e.g., “What’s your daddy’s name?”, “Are you married?”)^41^. In a longitudinal study of conversation logs with voice assistants, children (5–7 year-olds) showed persistent personification and emotional attachments to the technology^42^. Accordingly, one prediction is that adults will show larger distinctions in voice assistant and human registers than children, who will talk to the interlocutors more similarly.

On the other hand, *routinized interaction theories* propose that people develop ‘scripts’ for how to interact with technology that differ from how they engage with another person^43^. Technology-directed scripts are proposed to be based on real experience as well as a priori expectations (i.e., mental models) of how the systems understand them^43^. For example, adults rate text-to-speech (TTS) synthesized voices as ‘less communicatively competent’ than a human voice^15,22^. In the current study, a *routinization* prediction would be a consistent distinction for speech features in human- and technology-DS, such as those paralleling increased vocal effort in response to a communicative barrier (increased duration, pitch, and intensity in technology-DS). As mentioned, prior studies have found adults’ technology register adjustments are often louder^15,19,20^, have longer productions/slower rate^10,17,18,44^, and have differences in pitch^15,18,19,23,44^ from human-directed registers. Furthermore, a *routinization* prediction would be that, given their different experiences with systems, adults and children will vary in their device and human-directed registers. Children are misunderstood by automatic speech recognition (ASR) systems at a higher rate than adults^41,45–47^. For example, a voice assistant responded correctly to only half of queries produced by children (ages 5–10 years)^48^. In another study, speech produced by children (around age 5) was accurately transcribed only 18% of the time by the best performing ASR system^47^. Therefore, one possibility is that children will show more effortful speech patterns (increased duration and pitch) in voice-AI registers than adults, reflecting the expectation to be misunderstood, consistent with their interactions with voice assistants.

The current study compares English-speaking adults and school-age children (ages 7–12 years) in the United States in a psycholinguistic paradigm: a controlled interaction with a physically embodied human experimenter and Amazon Echo, matched in content, error rate, and error types. Prior studies employing fully controlled experiments with identical content and error rates for the human- and device-directed conditions often use pre-recorded voices and limited visual information (e.g., a static image of an Echo vs. a person)^10,15,17^. On the other end of the spectrum are studies that analyze speech from spontaneous interactions with physically embodied people and voice assistants^19,20,49^, but where the rate and type of errors are not controlled. In the current study, human experimenters in the current study followed written scripts to produce equivalent questions and responses as the Amazon Echo.

The human experimenter and Amazon Echo produced identical questions (e.g., “What’s number one?”), feedback (e.g., “I heard ‘bead’. Say the sentence one more time.”), and staged errors (e.g., “I think I misunderstood. I heard ‘bead’ or ‘beat’.”). This allows us to test overall speech adaptations, as well as adjustments to the local context: the participant’s first time producing a word^50,51^ compared to producing the word a second time after being correctly understood (less effortful)^52^, or after being misunderstood (more effortful)^51^. Prior work has shown few interactions between the context and adults’ register adaptations for voice assistants^15,17^, instead providing support for a more consistent set of acoustic adjustments (e.g., slower, higher pitch, smaller pitch range). At the same time, children might produce different local adjustments for technology-DS than adults. There are developmental differences in how children perceive^53^ and produce^54^ local adjustments. For example, when repairing an error made by a voice assistant (Alexa) in an interactive game, a vast majority of English-speaking preschoolers (ages 3–5) tended to increase their volume and roughly a third also tried different phrasing or pronunciation^55^. In a study with a computer avatar in a museum exhibit^56^, Swedish children (ages 9–12) tended to produce louder and more exaggerated speech in response to an error by the avatar, while adults tended to rephrase the utterance.

To probe human- and technology-DS registers, the current study examines two acoustic features: utterance duration and mean pitch (fundamental frequency, f0). If speakers' duration and pitch adaptations are identical for the two types of addressees, this would support *media equivalence*. However, if there are systematic differences in the way speakers tune their duration and pitch for technology than for a person, this would support *routinization*. In particular, we predict increases in duration and pitch for technology, paralleling adaptations for other communicative barriers (e.g., background noise^24,25^). Furthermore, we predict differences across adults and children in the current study based on both developmental and experiential differences with technology. If children show parallel duration and pitch adjustments for technology and people, this would support a developmentally-driven *media equivalence* account. Alternatively, if children show differences in duration and pitch to technology, relative to humans, this would support *routinization* accounts. Finally, we explore duration and pitch in response to addressee feedback: being correctly heard or misunderstood. If speakers show identical adjustments based on these local communicative pressures for Alexa and the human addressees, this would support *equivalence*, while distinct adjustments would support *routinization*. Responses to error corrections, additionally, can further shed light as to whether the types of adjustments made overall to technology reflect intelligibility strategies.

## Results

The acoustic measurements, analysis code, models, experiment code, and experiment video demo are provided in Open Science Framework (OSF) repository for the project (10.17605/OSF.IO/BPQGW).

### Acoustic adjustments by adults and children

**Figure 1:**
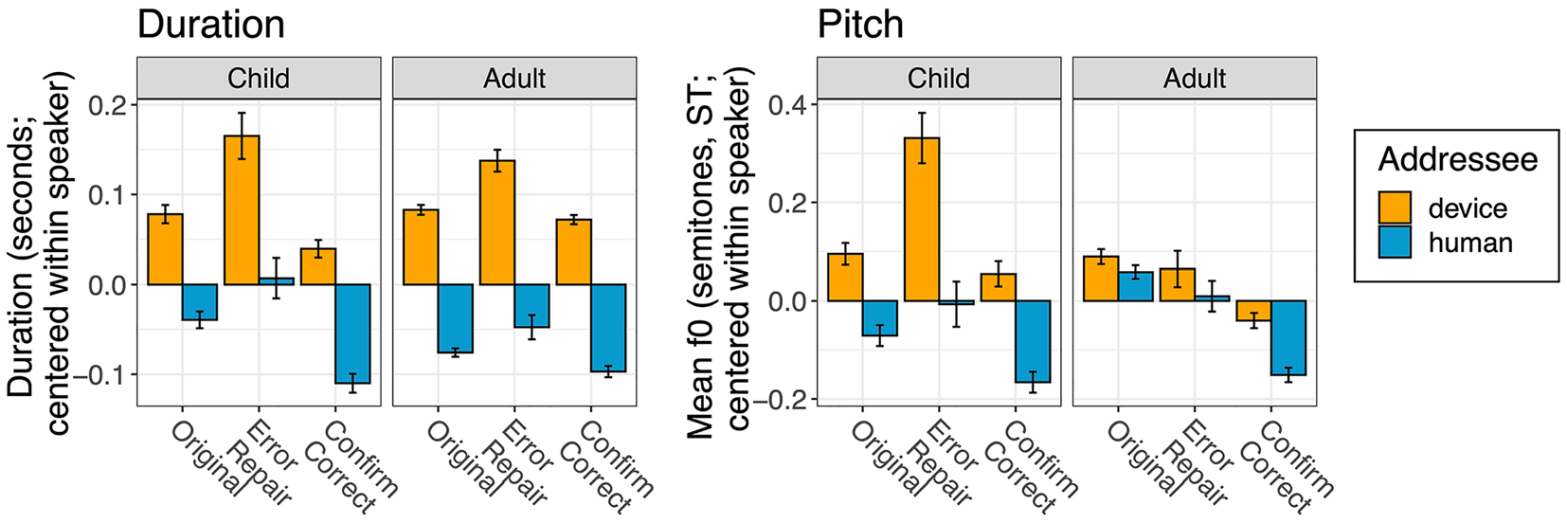
Prosodic changes from participants’ means in device- and human-directed utterances for adults and children for mean duration (left panel) and pitch (right panel) over the sentence, based on local communicative context: original, error repair, or confirm correct (x-axis). A value of “0” indicates no change from the speakers’ average, a negative value indicates a relative decrease, and a positive value indicates a relative increase.

**Figure 2:**
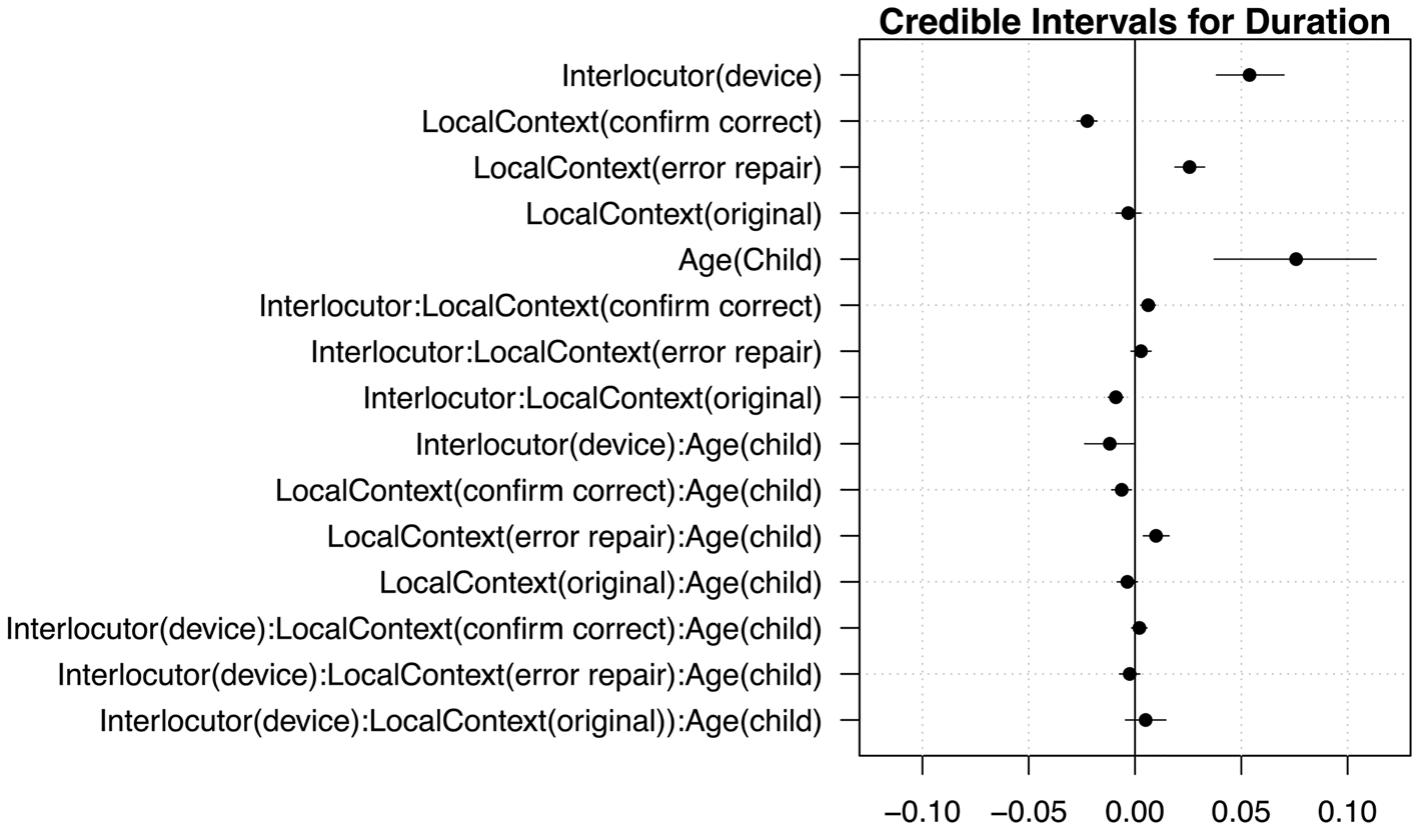
Credible intervals for the sentence duration model.

**Figure 3:**
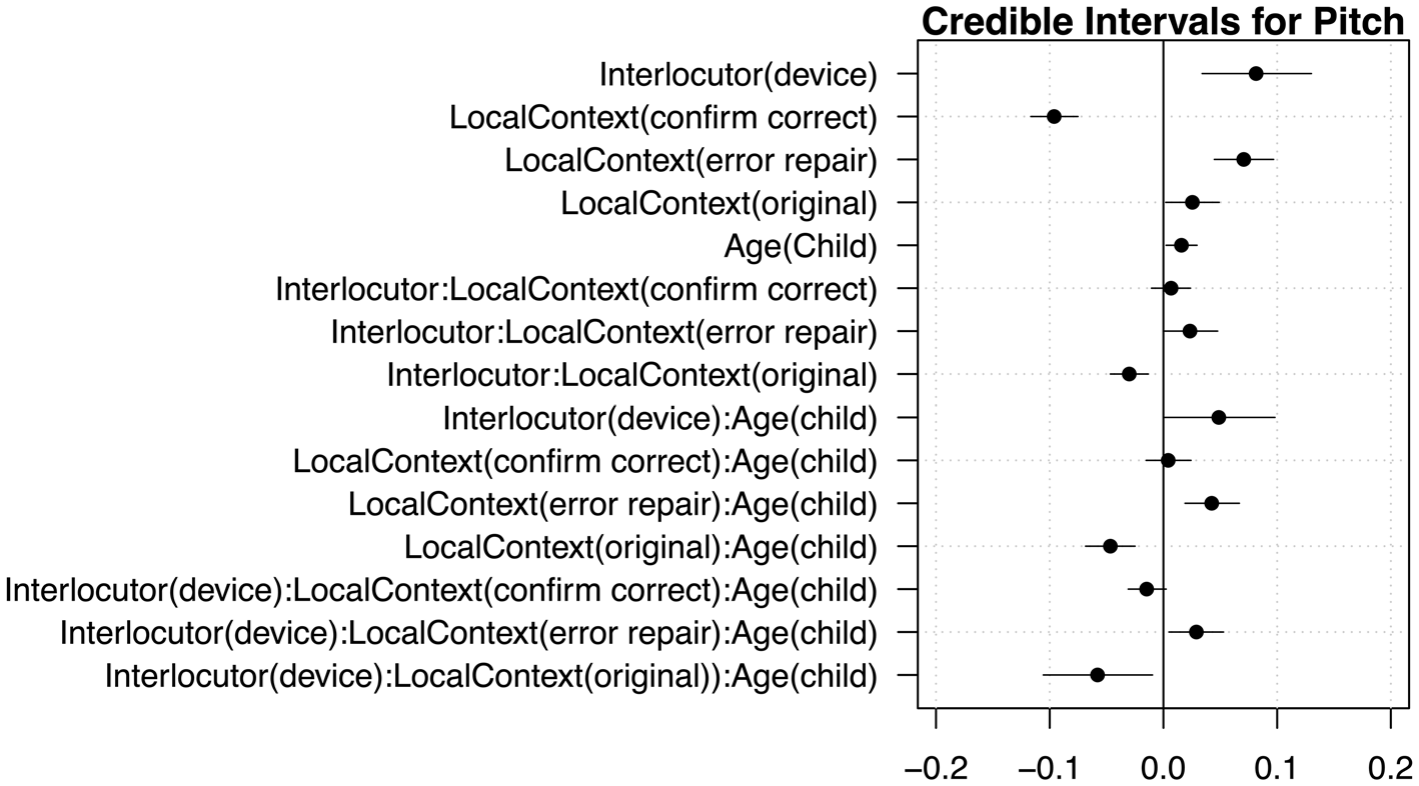
Credible intervals for the sentence pitch model.

Mean acoustic values across each condition are plotted in Fig. 1. Model outputs are provided in Tables 1 and 2, and credible intervals are plotted in Figs. 2 and 3. We report effects whose 95% credible intervals do not include zero or have 95% of their distribution on one side of 0.

**Table 1:** Model output for duration.

	Estimate	Est. error	Q2.5	Q97.5	% < 0	% > 0
**(Intercept)**	**0.41**	**0.02**	**0.36**	**0.45**	**0**	**100**
**Intercept(Sigma)**	**− 2.47**	**0.03**	**− 2.53**	**− 2.41**	**100**	**0**
**Interlocutor(Device)**	**0.05**	**0.01**	**0.04**	**0.07**	**0**	**100**
**LocalContext(ConfirmCorrect)**	**− 0.02**	**2.0e−03**	**− 0.03**	**− 0.02**	**100**	**0**
**LocalContext(ErrorRepair)**	**0.03**	**4.0e−03**	**0.02**	**0.03**	**0**	**100**
**Age(child)**	**0.08**	**0.02**	**0.04**	**0.11**	**0**	**100**
**Interlocutor(device):LocalContext(ConfirmCorrect)**	**0.01**	**2.0e−03**	**3.0e−03**	**0.01**	**0**	**100**
Interlocutor(device):LocalContext(ErrorRepair)	3.0e−03	2.0e−03	**− **2.0e−03	0.01	12	88
**Interlocutor(device):Age(child)**	**− 0.01**	**0.01**	**− 0.02**	**− 4.0e−05**	**98**	**2**
**LocalContext(ConfirmCorrect):Age(child)**	**− 0.01**	**2.0e-03**	**− 0.01**	**− 2.0e−03**	**100**	**0**
**LocalContext(ErrorRepair):Age(child)**	**0.01**	**3.0e−03**	**4.0e−03**	**0.02**	**0**	**100**
Interlocutor(device):LocalContext(ConfirmCorrect): Age(child)	2.0e−03	2.0e−03	**− **2.0e−03	0.01	13	87
Interlocutor(device):LocalContext(ErrorRepair): Age(child)	**− **2.0e−03	2.0e−03	**− **0.01	2.0e−03	85	15

Effects are in bold: credible intervals that have 95% of their distribution on one side of 0.

Num. observations = 10,867; Num. participants = 117; Num talkers = 10; Num. target words = 24.

**Table 2:** Model output for pitch (mean f0, centered within speaker).

	Estimate	Est. error	Q2.5	Q97.5	% < 0	% > 0
(Intercept)	0.02	0.02	− 0.02	0.05	18	82
**Intercept (Sigma)**	**− 0.74**	**0.03**	**− 0.8**	**− 0.68**	**100**	**0**
**Interlocutor(device)**	**0.08**	**0.02**	**0.03**	**0.13**	**0**	**100**
**LocalContext(ConfirmCorrect)**	**− 0.1**	**0.01**	**− 0.12**	**− 0.08**	**100**	**0**
**LocalContext(ErrorRepair)**	**0.07**	**0.01**	**0.04**	**0.1**	**0**	**100**
**Age(child)**	**0.02**	**0.01**	**0**	**0.03**	**1**	**99**
Interlocutor(device):LocalContext(ConfirmCorrect)	0.01	0.01	− 0.01	0.02	22	78
**Interlocutor(device):LocalContext(ErrorRepair)**	**0.02**	**0.01**	**0**	**0.05**	**3**	**97**
**Interlocutor(device):Age(child)**	**0.05**	**0.02**	**0**	**0.1**	**2**	**98**
LocalContext(ConfirmCorrect):Age(child)	0	0.01	− 0.02	0.02	34	66
**LocalContext(ErrorRepair):Age(child)**	**0.04**	**0.01**	**0.02**	**0.07**	**0**	**100**
**Interlocutor(device):LocalContext(ConfirmCorrect): Age(child)**	**− 0.01**	**0.01**	**− 0.03**	**0**	**96**	**4**
**Interlocutor(device):LocalContext(ErrorRepair): Age(child)**	**0.03**	**0.01**	**0**	**0.05**	**1**	**99**

Effects are in bold: credible intervals that have 95% of their distribution on one side of 0.

Num. observations = 10,867; Num. participants = 117; Num talkers = 10; Num. target words = 24.

First, the statistical models for both acoustic features revealed an effect of Interlocutor, where participants increased their utterance duration and pitch (mean fundamental frequency, f0) when talking to a device (here, Alexa) (see Fig. 1). Additionally, both models revealed effects of Local Context: if the addressee misheard them, participants increased their utterance duration and pitch when repairing the error. Conversely, if the addressee heard them correctly, participants decreased their duration and pitch when confirming.

The Local Context also interacted with Interlocutor: when confirming a correct reception, speakers produced even longer durations in device-directed speech (DS) (seen in Fig. 1, left panel). Additionally, when repairing an error, speakers produced even higher pitch in device-DS.

Additionally, there are the expected effects of Age Category, wherein children produce longer and higher pitched utterances overall. There were also interactions between Age Category and Local Context, wherein children tended to increase pitch and duration more in error repairs in general. Children also produced a shorter duration when confirming a correct reception (i.e., ‘confirm correct’) than adults.

Furthermore, the models revealed interactions between Age Category and Interlocutor: as seen in Fig. 1 (right panel), children produced even higher pitch in device-DS than when talking to a human experimenter (note that adults’ gender did not mediate this difference, see Supplementary Data, Table B). Additionally, children produced shorter utterances in device-DS; as this is sum coded, the converse is true: adults produced more consistently longer utterances in device-DS (seen in Fig. 1, left panel).

Finally, the pitch model revealed 3-way interactions between Interlocutor, Age Category, and Local Context. In device-DS, children produced an even larger increase in pitch to repair an error (seen in Fig. 1, right panel). At the same time, children showed a weaker pitch increase in device-DS when confirming a correct reception.

### Anthropomorphism responses by adults and children

**Figure 4:**
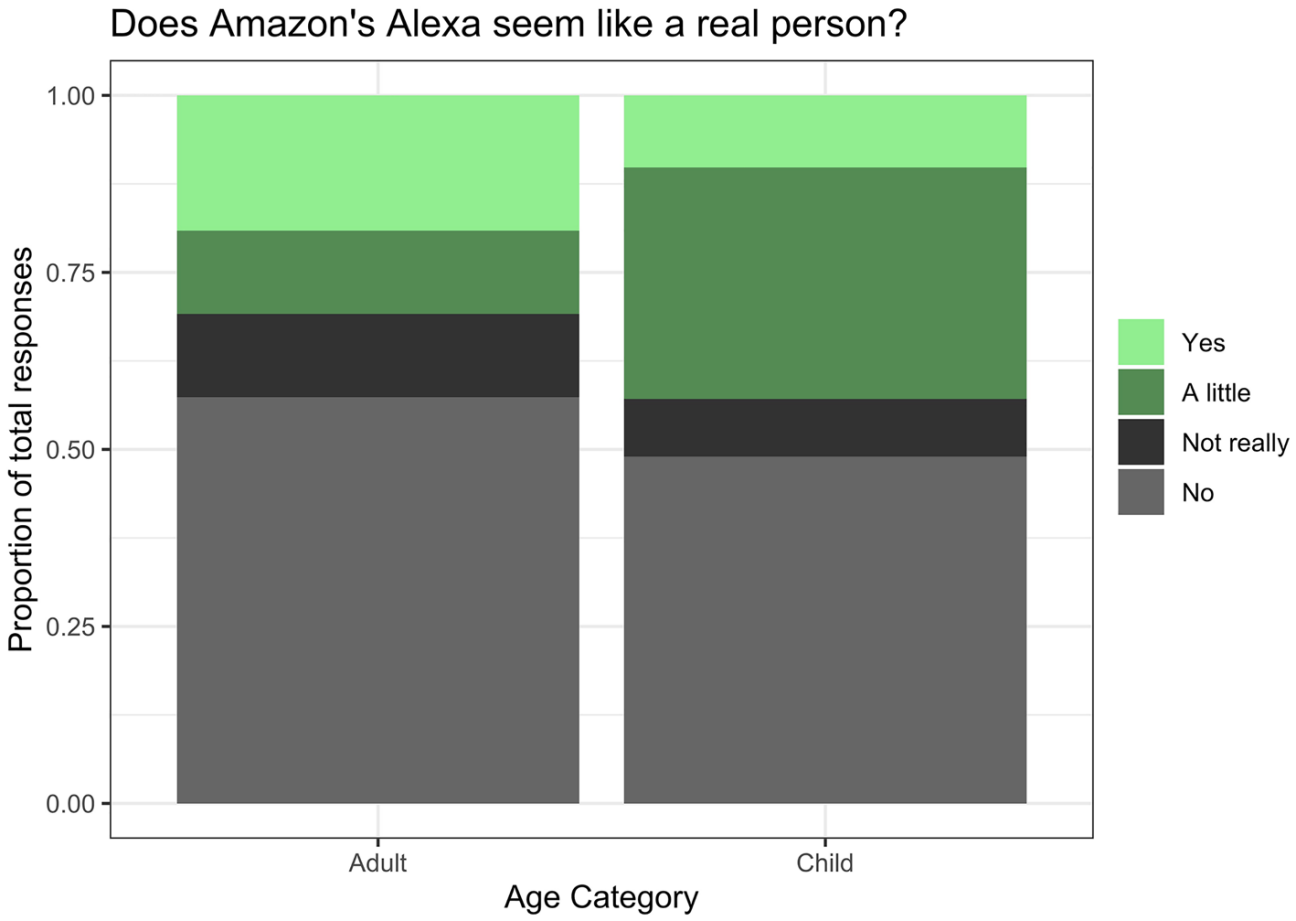
Proportion of responses for “Does Amazon’s Alexa seem like a real person?” for adult and child participants.

In response to the question asking if they thought “Alexa was like a real person” and to “explain why or why not”, adults and children both provided a range of responses, that we categorized as “yes”, “a little”, “not really”, or “no”. While there was variation, as seen in Fig. 4, the ordinal logistic regression model showed no difference between the age groups in their response distributions [*Coef* = 0.18, *SE* = 0.63, 95% CI (− 1.04, 1.54)], suggesting a similar degree of overall anthropomorphism.

### Post hoc: technology adjustments mediated by anthropomorphism?

In order to test whether adults’ and children’s device-DS register adjustments were driven by their anthropomorphism of the Alexa system, we included Anthropomorphism as a predictor in the duration and pitch models. Both the duration and pitch models showed no simple effect of Anthropomorphism, but two interactions between Anthropomorphism and other predictors (credible intervals both 95% below 0). The duration model showed one interaction between Interlocutor, Local Context, and Anthropomorphism [*Coef* = − 0.03, *SE* = 0.02, 95% CI (− 0.06, 0.01)]: for individuals who tended to anthropomorphize, there was less of an increase in duration in device-DS confirming correct responses (‘confirm correct’). The pitch model showed an interaction for Local Context and Anthropomorphism [*Coef* = − 0.01, *SE* = 4.7e−03, 95% CI (− 0.02, − 1.4e−03)], with a lower pitch in ‘confirm correct’ overall for individuals with higher anthropomorphism scores.

## Discussion

The current study used a psycholinguistic paradigm to compare voice-AI and human-directed registers, using authentic, physically embodied human and smart speaker addressees in a controlled experiment. This approach extended prior studies that used pre-recorded voices^15^ or non-controlled interactions (e.g., containing ASR errors)^19,20^. Additionally, we compared a cross-section of ages (adults vs. school-age children) to probe both developmental and experiential factors that could shape speech adaptations toward technology.

We found that both adults and children produced adaptations in device-directed speech (DS), compared to when talking to another person. Device-DS had longer and higher pitched utterances overall. These adjustments replicate a related study comparing Alexa- and human-DS in a similar paradigm that found a slower rate and higher pitch in device-DS by English speaking college-age participants, but that used pre-recorded voices and had a much higher error rate (50%) compared to the current study (16.7%)^17^. A higher pitch has only been reported for two other studies for device-DS, one in German (voice assistant)^19^ and one in French (robot)^23^. Duration increases (or decreased speech rate) is a more commonly reported feature of technology-DS for adults (e.g., for a computer avatar^10^ or imagined computer^44^, or Alexa socialbot^16^, or social robot^21^). In the current study, adults and children made both duration and pitch adjustments, supporting *routinized interaction theories* of human–computer interaction^43^, in which people have distinct modes of engaging with technology than with other humans.

The device-DS adjustments appear to be in an effort to improve intelligibility for an addressee facing communicative barriers. For example, in related work, speakers have been shown to increase duration and pitch in the presence of background noise^25^. In the current study, we found that speakers also increased duration and pitch when repairing an error; when communication went smoothly, they decreased both of these features. Indeed, prior work has shown that college-age adults rate voice-AI as being less communicatively competent than human interlocutors^11,15^. Consistent with this interpretation, we also see that even when Alexa correctly heard them, speakers maintained duration increases. This is in contrast to second mention effects^52^, but parallels related work, such as maintaining a higher pitch in second mentions for infant-DS^57^.

The age of the speaker is also an important factor in how a voice-AI register was realized in the current study. In particular, children (here, ages 7–12) showed larger increases in pitch when talking to Alexa compared to when talking to a person. Children also increased their pitch even more for Alexa in response to an apparent ASR error. While one prediction was that children would show greater *media equivalence*, given their tendency to anthropomorphize non-human entities^36,37^, we instead see that children demonstrate a systematized set of acoustic adjustments when talking to technology. These adjustments are even more pronounced in the local contexts: children increased pitch even more after Alexa misunderstood them, and decreased it more when Alexa heard them correctly, suggesting that pitch is part of children’s effortful and intelligibility-related adjustments for technology. Taken together, we interpret children’s consistent pitch and duration adjustments as stemming from their experience being misunderstood by ASR systems^46,47^, supporting *routinized interaction accounts*^43^.

While children tended to target both pitch and duration in device-DS, adults tended to prioritize longer duration. Overall, adults made smaller changes in pitch across the addressees (Alexa, human) and local contexts (e.g., confirm correct, error repair). This finding suggests one possible explanation for why prior studies examining adults’ adaptations to technology tend to not observe pitch increases^10,21^. Using pitch as a strategy to improve intelligibility might only come into play when the error rate is high; as mentioned, in the related study that found slower rate and higher pitch by adults to a pre-recorded Alexa voice, the error rate was higher (50% of trials)^17^. The shift away from pitch adjustments as a primary intelligibility strategy might also reflect children’s development in social cognition. For example, we found that children used both higher pitch and duration in correcting errors made by the *human* as well (though this was more pronounced in device-DS). This pattern is consistent with related work showing that children use distinct strategies to improve intelligibility than adults; when misunderstood by technology, both young children (ages 3–5) and school-age children (ages 9–12) tend to increase their volume, while adults tend to rephrase the utterance^56^. Taken together, adults’ and children’s differing adjustments reflect how they conceive of their addressee’s barrier and their strategy to overcome it.

In addition to probing speech behavior in the interactions, we examined participants’ responses to the question “Does Alexa seem like a real person?”. We found that adults and children provided parallel distributions in responses; roughly half of adults and children indicated some anthropomorphism (responding “somewhat” or “yes”). Furthermore, anthropomorphism did not mediate the overall register adjustments in device-DS (longer duration, higher pitch). We do see evidence for one context-specific difference for device-DS: individuals who demonstrated anthropomorphism also tended to produce more similar second mention reduction effects for Alexa and the human addressees. While speculative, it is possible that *media equivalence*^26–28^ might shape the local communicative pressures (e.g., being heard correctly) more so than the overall register characteristics. When a person believes a system to be more human-like *and* communication goes smoothly, will we see greater *media equivalence*? Future work examining individual variation in anthropomorphism in register adaptation studies are needed to test this possibility.

Broadly, these findings contribute to the wider literature on addressee adaptations (e.g., ‘Audience Design’^12–14^), such as infant-^6,7^, non-native speaker-^8,9^, hard-of-hearing-^58,59^, and pet-DS^60,61^ registers. In some ways, the increase in duration and pitch parallel adaptations made for infants. Infant-DS is also characterized by slower rate (and longer duration), higher pitch, and wider pitch variability. Do adults and children talk to technology more like an infant, believing it to also be a language learner? Related work suggests the adaptations might not be equivalent; for example, adults produce less pitch variation in technology- than human-DS in some studies^15,18^ and rate voice assistants as having adult ages^18,62^. Additionally, the motivations in IDS and technology-DS likely vary; related work has shown less emotional affect in non-native-speaker-DS than IDS^8^ and similarly less affect proposed in technology-DS^10^. Future work probing directly comparing multiple registers (e.g., infant-, non-native-speaker, technology-DS) are needed to better understand the motivations across register adaptations.

This study has limitations that can serve as directions for future research. First, our sample of English-speaking college-age adults and school-age children from California serves as a slice of the world’s population. Recent work has highlighted the differences in ASR for non-native speakers^63^ and speakers of other dialects (e.g., African American English^64,65^). The extent to which routinization for technology-DS is even stronger for speakers more commonly misunderstood by voice technology is an avenue for future work.

Furthermore, children in the current study ranged from ages 7–12. Prior work has suggested that children’s conceptualizations of different speaking styles appear to develop even earlier. For example, three-year-olds produce adult- and infant-directed registers (e.g., in doll playing^66^) and preschoolers show distinctions in speech in difficult listening conditions^67^. Therefore, it is possible for younger children to develop *routinized* technology-DS registers. At the same time, developmental differences in theory of mind^68^, or the ability to infer another’s point of view, can emerge as early as the age of two^69^. While speculative, the ability to adapt speech in anticipation of another person’s real (or assumed) communicative barriers, then, might also develop in tandem. Future research examining other child age groups and tracking an individual child’s behavior over the course of development^42^, particularly in light of individual variation in children’s anthropomorphism^70,71^, are needed for a fuller picture of conceptualizations of technology across development.

While intensity (related to perception of loudness) has also been identified as a feature of technology-DS registers in prior work^15,19,72^, the current study was limited by the Zoom settings for the interaction, wherein intensity was normalized to 70 dB by default. As the experiment was conducted during the COVID-19 pandemic, in-person experiments with head-mounted microphones were not possible. However, our approach does allow for future analysis of multimodal speech behaviors in the recorded interactions (e.g., gestural increases in speech produced in noise^73,74^). A Zoom-mediated interaction also provides a slightly more naturalistic interaction where participants could expect an adult person to mishear them (as they do in 16.7% of trials), compared to in a sound-attenuated booth where such errors would be less expected. Future studies with in-lab experiments, and using head-mounted microphones, is needed to explore the role of intensity, as well as to probe the consistency of the technology-DS adjustments across contexts.

As mentioned in the Introduction, a growing body of work has shown that people perceive socio-indexical properties of TTS voices as well, such as age and gender. Here, we held the gender of both the human and TTS voices constant (all female). This was to maximize the number of possible voice options (at the time of the study, Amazon Polly had 4 US-English female voices, but only 2 male voices available), and we recruited six female research assistants to provide comparable variation in the human voices. Each participant was exposed to just one TTS and one human addressee. Future work examining more variation in the types of voices (e.g., ages, genders, dialects) can shed light on additional social factors mediating human–computer interaction.

Moreover, while this study provided methodological advancements in examining how people adapt their speech to a human and device, it is limited to a single sociocultural and linguistic context: native English speakers in the United States (specifically in California). This limitation raises avenues for future study examining perception of human and technology interlocutors across dialects and languages.

For example, German-speaking children (ages 5–6 years), slightly younger than those in the present study, produce larger increases in pitch and intensity when talking to an apparent human than voice assistant in a Wizard-of-Oz experiment^75^. While a growing area of study, there are also cross-cultural attitudes about technology^76^ that could further shape their conceptualization as ‘human’ or ‘machine’. Finally, access to technology is not equitable for people worldwide. The vast majority of the world’s ~ 7000 languages are not supported by digital language technology^77,78^. Future work examining different cultural attitudes, anthropomorphism, and language technology acceptance are needed for a comprehensive test of human cognition in an increasingly technological world.

## Methods

### Participants

A total of 89 adult participants were recruited from the UC Davis Psychology subjects pool and completed the study. Data was excluded for n = 19 participants, who had technical difficulties (e.g., slow Wi-Fi; n = 11), reported hearing impairments (n = 3), who had consistent background interference (n = 1), or were non-native English speakers (n = 4). Data was removed for n = 2 participants who had an extra staged error for one addressee (an experimental coding error). The retained data consisted of 68 adults (mean age = 19.96 years, sd = 3.34, range = 18–44; 33 female, 35 male). All participants were native English speakers from California, with no reported hearing impairments. Nearly all participants reported prior experience with voice-AI (n = 31 Alexa; n = 47 Siri; n = 19 Google Assistant; n = 5 other system; n = 3 reported no prior usage of any system). This study was approved by the Institutional Review Board (IRB) at the University of California, Davis (Protocol 1407306) and participants completed informed consent. Participants received course credit for their time.

A total of 71 child participants (ages 7–12) were recruited from parent Facebook groups and elementary school listservs across California and completed the study. Due to technical difficulties, data was excluded for n = 6 participants. Data for n = 10 children was also excluded as they had difficulty completing the study (e.g., pronouncing the words, background noise). Data was removed for n = 6 participants who had an extra staged error for one interlocutor. The retained data consisted of 49 children (mean age = 9.55 years, sd = 1.57; 27 female, 20 male, 2 nonbinary). All children were native English speakers from California, with no reported hearing impairments. Nearly all children reported prior experience with voice-AI (n = 35 Alexa; n = 34 Siri; n = 24 Google Assistant; n = 3 other system; n = 1 reported no prior usage of any system). This study was approved by the Institutional Review Board (IRB) at the University of California, Davis (Protocol 1407306) and children’s parents completed informed consent while the child participants completed verbal assent. Children received a $15 gift card for their time.

### Stimuli

We selected 24 CVC target words with an age-of-acquisition (AoA)^79^ rating under 7 years (mean = 4.77, sd = 1.01; range = 2.79–6.68), with the exception of one common name (“Ben”). All words had a final voiced coda: either a voiced oral stop (e.g., “seed”) or a voiced nasal stop (e.g., “shine”). Target words were selected to have a final coda or nasal minimal pair (e.g., “seed” ~ “seat”; “Ben” ~ “bed”) for the staged error conditions (by the human or Alexa interlocutor), paralleling the approach of related studies comparing human- and device-DS^15^. A full list of target words is provided in Supplementary Data, Table A.

### Procedure

Participants signed up for a timeslot on a centralized online calendar for the project, Calendly, and were randomly assigned to an available experimenter for that time (generating a unique Zoom link for the interaction). All participants completed the experiment remotely in a Zoom video-conferencing appointment with a trained undergraduate research assistant (n = 6; all female native English speakers, mean age = 21.5 years; range: 19–25). Each of the 6 experimenters had a set-up that included the identical Amazon Echo (3rd Generation, Silver) and TONOR omnidirectional condenser microphone array (to control for audio input across their computer systems). Experimenters additionally had an Alexa ‘App’ on their smartphones and logged into the same lab account to access versions of the Alexa Skills Kit app. Before the interaction, experimenters set the Echo volume level to ‘5’ and put the device on ‘mute’ until the Device interlocutor block.

**Figure 5:**
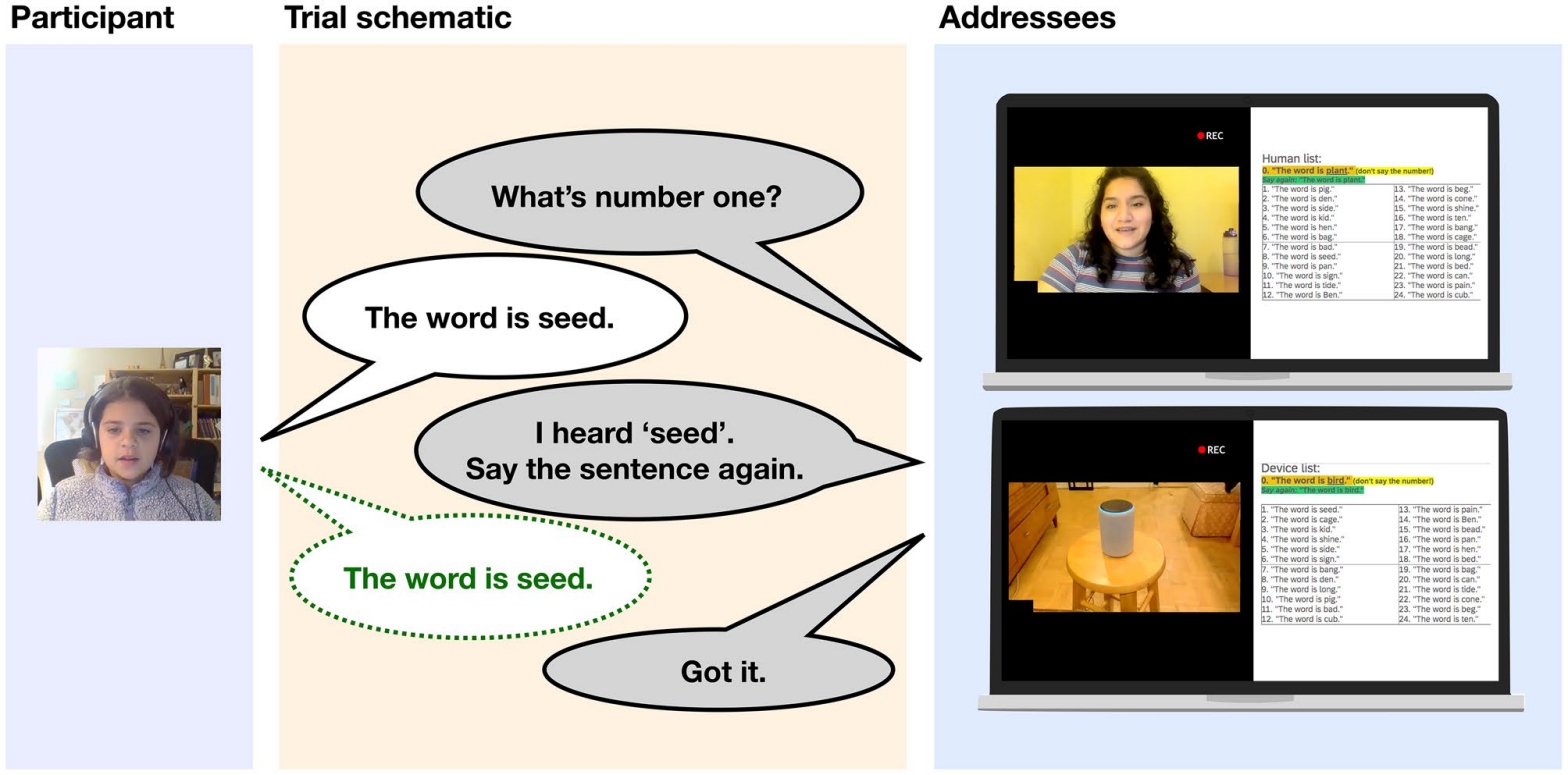
Experiment schematic for each trial. Each trial consisted of five turns. First, the interlocutor asks what the word is for number one. The participant read the appropriate sentence from the list from the Qualtrics website (first mention), heard feedback from the interlocutor, and read the sentence again (second mention, shown in dashed green). Finally, the interlocutor responded with a closing statement (e.g., “Got it”, “Alright”, etc.). Participants completed the interaction with both the experimenter and the Alexa Echo (order counterbalanced across participants). Note that the child’s guardian consented to the use of the child participant’s image in an Open Access article. Additionally, the research assistant (addressee) consented to the use of her image in an Open Access article.

At the beginning of the session, the experimenter sent a Qualtrics survey link in the Zoom chat to the participant and read instructions using a script to direct participants how to set up their screens (with the Zoom video partitioned to the left-hand half and the Qualtrics survey partitioned to the right-hand half) (shown in Fig. 5).

Participants completed two interaction blocks of the experiment: one with the experimenter as the interlocutor, one with the device as the interlocutor (shown in Fig. 5; order of interlocutor blocks counterbalanced across participants). At the beginning of each block, the interlocutor (human or device) gave spoken instructions for the task (provided in OSF Repository).

### Voice assistant interlocutor

For the voice assistant block, a transcript of the interaction including all instructions, pauses for subjects’ responses (5 s; using <break time> SSML), and interstimulus intervals (1.5 s) were generated as input for the TTS output in two Alexa Skills Kit applications. In each, one of 4 US-English female Amazon Polly voices (‘Salli’, ‘Joanna’, ‘Kendra’, or ‘Kimberly’) was randomly selected. After the RA engaged the skill, it continuously produced TTS output (e.g., “What’s number 1? <break time = ‘5 s’> </break> I heard, seed. Say the sentence one more time. <break time = ‘5 s’> </break> Great <break time = ‘1.5 s’> </break>”) to avoid ASR errors. The experimenter opened the device interlocutor by unmuting the Echo and saying ‘Alexa, open Phonetics Lab Zoom study’ (Version A) or ‘Alexa, open Phonetics Lab version B’ (Version B).

### Human interlocutor

For the human interlocutor block, the experimenter followed a Qualtrics experiment with script (provided in OSF repository). In experimental trials, the researcher read each sentence, and saw a 5 s countdown to match the planned pause time in the Alexa output.

### Sentence lists

For each interlocutor, there was a corresponding Sentence List provided on the Qualtrics survey: one labeled for ‘device’ and one for ‘human’ (correspondence was counterbalanced across participants). In each Sentence List, there were 24 target words, which occurred phrase-finally in the sentence frame (“The word is ___”). Each Sentence List had 4 versions (randomly selected), which pseudorandomized the interlocutor’s response and final feedback, and varied which sentences the errors occurred on. Occurrence of the interlocutors’ staged errors was controlled: two voicing errors and two nasality errors occurred roughly equally throughout the interaction (every 5–6 trials), with the first error occurring within the first 6 trials. In both the human and Alexa interlocutor blocks, the error rate was 16.7% (4/24).

### Experimental trials

On each trial, there were five fully scripted turns, illustrated in Fig. 5. First, the interlocutor asked “What’s number 1?”. Next, the participant read the corresponding sentence on their human/device list. The interlocutor then responded: either with certainty and responding with the correct word (“I heard pig”) or with uncertainty and responding with an incorrect distractor item (incorrect voicing or nasality) and the target word (“[I missed part of that|I didn’t catch that|I misunderstood that]. I heard pick or pig”). Next, the interlocutor asked the subject to repeat the sentence (4 phrase options, pseudorandomized across trials: “Say the sentence one more time”, “Repeat the sentence another time”, “Say the sentence again”, “Repeat the sentence one more time”). The subject then produced the sentence again. The trial interaction ended with the interlocutor responding with a final response (“Alright.”, “Got it.”, “Thanks.”, “Okay.”) (pseudorandomized).

### Data annotation

The interactions were initially transcribed using the native Zoom speech recognition (based on Sonix ASR), which separated the experimenter and participant streams based on the Zoom interaction. Trained undergraduate research assistants listened to all experiment sessions, and corrected the ASR output and annotated the interaction in ELAN^80^ by (1) indicating portions of the researcher stream as ‘human’ and ‘device’ for the experimental trials, (2) indicating presence of staged misrecognitions, and (3) indicating presence of unplanned errors or background interference (e.g., Zoom audio artifact; lawnmower sound; parent talking). We excluded 69 trials where there was background noise (e.g., dog barking, another person talking, motorcycle noise), 163 trials with a technical issue (e.g., internet glitch, audio inaudible), 241 trials with a mispronunciation or false start (e.g., read the wrong word, mispronounced the target word), 22 trials where there was overlap between the participants’ speech and either the experimenter or Echo, and 77 other errors. The retained data consisted of n = 49 children, and n = 68 adults, with 10,867 observations for the experimental trials.

### Acoustic analyses

Mean acoustic measurements were taken over each target sentence in Praat^81^. We measured utterance duration in milliseconds and logged the values. For pitch, we measured mean fundamental frequency (f0) (averaged over 10 equidistant intervals^82^ to get a more stable measurement^15^). We measured f0 for adult male, adult female, and child speakers separately, using plausible maxima and minima (adult males: 78–150 Hz; adult females: 150–350 Hz; children: 150–450 Hz) and converted the values to semitones (ST, relative to 75 Hz).

### Statistical analyses

We modeled participants’ acoustic properties of interest (duration, pitch) from experimental trials in separate Bayesian mixed effects regression models using the *brms*^83^ implementation of *Stan*^84^ in R^85^. Each model included effects of Interlocutor (device, human), Local Context (original, error repair, confirm correct), Age Category (adult, child) and all possible interactions. Factors were sum coded. We also included random intercepts for Talker, Word, and Participant, as well as by-Participant random slopes for Interlocutor and Local Context. We also included by-Participant random intercepts for the residual error (sigma) to account for differences in the residual for each speaker, as well including a fixed effect for sigma. We set priors for all parameters for each acoustic property based on values from a related experiment^15^.

### Anthropomorphism

At the end of the experiment, participants were asked “Does Alexa seem like a real person? Why or why not?”). A full list of participants’ responses is provided in the OSF Repository. We coded responses as ordinal data (“No” < “Not really” < “A little” < “Yes”), and analyzed responses with an ordinal mixed effects logistic regression with the *brms* R package^83^. Fixed effects included Age Category (child, adult; sum coded).

### Post hoc: anthropomorphism and register adaptations

We coded participants’ responses as to whether “Alexa seems like a real person or not” as binomial data (= 1 “no” or “not really”, = 0 if not) (full set of responses available in the OSF repository). We modeled participant’s utterance (log) duration and pitch (mean f0) in separate linear regression models with *brms*^83^, with the same model structure as in the main analysis, with the additional predictor of Anthropomorphism (2 levels: higher, lower), and all possible interactions.

### Ethics and consent

All research methods, including informed consent and child assent, were performed in accordance with the relevant guidelines and regulations of Protocol 1407306 of the Institutional Review Board (IRB) at the University of California, Davis.

### Supplementary Information

Supplementary Information.

## Data Availability

The data that support the findings of this study, including full model outputs, are openly available in an Open Science Framework (OSF) repository for the paper at 10.17605/OSF.IO/BPQGW.
