# Supplementary material for: Children and adults produce distinct technology- and human-directed speech
Source: Sci Rep. 2024 Jul 6;14:15611. doi: 10.1038/s41598-024-66313-5 (PMC11227501; doi:10.1038/s41598-024-66313-5)
Supplement: Supplementary file 1 — Supplementary Information. [file 41598_2024_66313_MOESM1_ESM.pdf]

Table A. Target Word Stimuli

	Target word	AoA		Target word	AoA
1	ten	3.58	13	bad	2.79
2	long	4.24	14	bed	2.89
3	pain	4.26	15	pig	3.84
4	can	4.32	16	bag	4.28
5	sign	4.32	17	kid	4.28
6	cone	4.67	18	side	4.72
7	pan	4.72	19	seed	4.72
8	den	5.7	20	cage	5.06
9	shine	5.79	21	cub	5.40
10	hen	6.39	22	bead	5.63
11	Ben	*N/A*	23	beg	5.78
12	bang	5.74	24	tide	6.68

Table B. Post hoc analysis of pitch by speaker age and gender

To test whether the increases in device-DS between child and adult speakers was similar across adult women and men, we conducted a post hoc analysis, with an AgeGender categorical variable (sum coded): adult(male), adult(female), and child. The model showed the Interlocutor effect: higher pitch in device-DS [Coef = 0.06, 95% CI(-0.04, -3.8e-03)], but there were no interactions between Interlocutor*AgeGender for the adults: Interlocutor(device)*AgeGender(adult-F): [Coef = -0.03, 95% CI (-0.09, 0.04)] and Interlocutor(device)*AgeGender(adult-M): [Coef = -0.03, 95% CI (-0.1, 0.04)]. This suggests that the weaker pitch increases for adults, relative to children, is not mediated by the adults' gender.
